# Complete Genome Sequence of Nervous Necrosis Virus Isolated from Sevenband Grouper (*Epinephelus septemfasciatus*) in South Korea

**DOI:** 10.1128/genomeA.01264-14

**Published:** 2014-12-11

**Authors:** Jong-Oh Kim, Wi-Sik Kim, Jae-Kwon Cho, Kyong-Min Kim, Maeng-Hyun Son, Myung-Joo Oh

**Affiliations:** a Department of Aqualife Medicine, Chonnam National University, Yeosu, Republic of Korea; b Southeast Sea Fisheries Research Center, National Fisheries Research and Development Institute, Yeosu, Republic of Korea

## Abstract

The draft genome sequence of the nervous necrosis virus (NNV) SGYeosu08, isolated from sevenband grouper (*Epinephelus septemfasciatus*) in Yeosu, South Korea, was cloned and analyzed. The full-length RNA1 was a 3,103-nucleotide-encoding region of RNA-dependent RNA polymerase, and the RNA2 encoding a coat protein was 1,433 nucleotides in length. This genome sequence might be useful in the development of an accurate diagnostic tool.

## GENOME ANNOUNCEMENT

Viral nervous necrosis (VNN), also known as viral encephalopathy and retinopathy (VER), is a serious viral disease in more than 20 species of cultured marine fish worldwide (1–4). VNN-affected fish commonly show abnormal swimming behavior and vacuolization and necrosis of the central nervous system and the retina (5). VNN has a high mortality rate (80 to 100%) in hatchery-reared larvae and juveniles in a number of species (5). Nervous necrosis virus (NNV), the etiological agent of VNN, is a small nonenveloped virus and belongs to the family *Nodaviridae*. It has two positive-sense single-strand RNAs as a viral genome. RNA1 is approximately 3.1 kb in length and encodes a protein A, RNA-dependent RNA polymerase (RdRp), while RNA2 is about 1.4 kb in length and encrypts a protein B, coat protein. Nodaviruses are divided into 4 genogroups, based on the sequence of the T4 region in RNA2, barfin flounder nervous necrosis virus (BFNNV), red-spotted grouper nervous necrosis virus (RGNNV), striped jack nervous necrosis virus (SJNNV), and tiger puffer nervous necrosis virus (TPNNV) (6).

The NNV isolated from sevenband grouper cultured in Yeosu, South Korea, at 2008 (SGYeosu08) and propagated in the snakehead fish cell line (SSN-1). The nucleotide sequences of NNV were amplified by reverse transcription-PCR (RT-PCR). Then, the PCR products were purified with a QIAquick gel extraction kit (Qiagen) and ligated with the pGEM-T easy vector system (Promega). All transformants were confirmed by restriction enzyme digestion and the clones sequenced with an ABI 3730 XL DNA sequencer. All analyzed sequences were assembled and analyzed manually.

The RNA1 segment is composed of 3,103 nucleotides and contains an open reading frame (ORF)-encoding RdRp, which is located between 79 and 3,027 nucleotides (2,949 bp) from the 5′ end of the genomic RNA. RdRp is composed of 982 amino acids, with a calculated molecular mass of 110.1 kDa. The RNA2 segment is composed of 1,433 nucleotides and contains an ORF-encoding coat protein, which is located between 26 and 1,042 nucleotides (1,017 bp). Coat protein is composed of 338 amino acids, with a calculated molecular mass of 37.1 kDa. This genome information may be useful in the development of accurate and rapid diagnostic tools and reverse genetic approaches.

### Nucleotide sequence accession numbers.

The complete genome sequence of the NNV isolate SGYeosu08 has been deposited in GenBank under the accession numbers KM095958 and KM095959.
